# The impact of leisure sedentary behaviors on risk of chronic kidney disease, diabetes, and related complications: Mendelian randomization study

**DOI:** 10.1080/0886022X.2025.2479177

**Published:** 2025-03-20

**Authors:** Shuo Zhong, Rui Xiao, Ying Lin, Bo Xie, Jing Sun

**Affiliations:** ^a^ Department of Nephrology, Shandong Provincial Hospital, Shandong University, Jinan, China; ^b^ Department of General Practice, Yongchuan Hospital of Chongqing Medical University, Chongqing Medical University, Chongqing, China; ^c^ Jinan Center for Disease Control and Prevention, Jinan, China

**Keywords:** Mendelian randomization, chronic kidney disease, diabetes mellitus, type 1 diabetes, type 2 diabetes, complications of diabetes

## Abstract

**Background:**

The causal relationship between leisure sedentary behaviors (LSBs) and chronic kidney disease, diabetes and related complications is still equivocal. In this study, we performed two-sample Mendelian randomization for declaring the potential causal association between LSBs and these diseases and summarized the causal estimates.

**Methods:**

In this study, we used GWAS summary statistics from the public database for exposures (LSB: television watching, computer use, and driving) and outcomes (chronic kidney diseases, diabetes mellitus, and related complications). To ensure reliable results for this study, we applied several methods including IVW, MR-Egger, and weighted median for the regression process; MR-Egger intercept test, Cochran’s Q test, ‘leave-one-out’ analysis and MR-PRESSO test were used to detect horizontal pleiotropy and heterogeneity for sensitivity analysis.

**Results:**

Television watching was harmful of CKD (OR = 1.26, 95%CI 1.09–1.44; *p* = 0.0011), T2D (OR = 1.82, 95%CI 1.48–2.24; *p* = 1.67e − 08) and DM (OR = 2.26, 95%CI 1.75–2.93; *p* = 6.44e − 10). No horizontal pleiotropy was detected in MR-Egger intercept test (*p* value > 0.05) and there were no influential SNPs based on ‘leave-one-out’ analysis.

**Conclusions:**

Mendelian randomization estimates in our study genetically predicted the causal effect between television watching and CKD, T2D, and DM. However, we cannot get the definitive causal effect of television watching and other related complications, further studies need to be done to explore the mechanism of action of sedentary behavior on the complications of diabetes and chronic kidney disease.

## Background

Chronic kidney disease (CKD) will be the fifth and diabetes will be the seventh highest cause of years of life lost (YLLs) worldwide by 2040 [1]. Meanwhile, three metabolic risks—high blood pressure, high body mass index (BMI), and high fasting blood glucose (FPG)—as among the five leading global risk factors for YLLs in 2040 [1]. It is estimated that by 2017, more than 23 million people worldwide have died of unhealthy behaviors, accounting for 36.5% of the disability-adjusted life years [2]. In addition, many organizations such as the World Health Organization have approved relevant policies and pointed out that adopting healthy lifestyle behaviors is the most cost-effective strategy to prevent noncommunicable diseases [3].

Due to a variety of modern technological advancements, including computer hardware, task automation facilitated by increasingly sophisticated software, an increase in the forms and numbers of electronic communication modes, and individually dedicated office equipment and furniture [4], nowadays, it is acknowledged that leisure sedentary behavior (LSB) is a potential health threat [5].

Sedentary behavior is any waking behavior characterized by an energy expenditure ≤1.5 metabolic equivalents (METs), while in a sitting, reclining, or lying posture [6]. Prolonged sedentary behaviors, have been shown to contribute to metabolic disturbances such as insulin resistance and adiposity, which increase the risk of chronic kidney disease (CKD), type 2 diabetes (T2D), and diabetes mellitus (DM) [7–9]. Particularly in cross-sectional studies, a sedentary lifestyle was positively and independently correlated with a number of variables linked to impaired kidney function [10]. Unhealthy lifestyles such as sedentary behaviors (including television watching, computer use, and driving) can lead to fat remodeling, further leading to obesity, and eventually lead to type 2 diabetes (T2D) and adverse cardiovascular disease outcomes [11,12]. Observational studies commonly utilize television watching as a surrogate for overall LSB because it is primarily done in work environments and can be changed with intervention [13–15]. Also, as television watching is easier to remember, it has a higher validity than engaging in entirely sedentary behavior [16]. However, direct evidence of the causal effects of sedentary behavior on CKD, diabetes mellitus (DM), and related complications remain unclear.

Large-scale randomized controlled trials (RCTs) can be used to explore the relationship between sedentary behavior and diseases. However, RCTs are costly, labor-intensive, time-consuming, and have ethical constraints, making them difficult or impractical to conduct [17].

As an alternative, Mendelian randomization (MR), a natural RCT, is a widely used, more practical method for determining the causal relationship between an exposure and an outcome [18].

In the MR analysis, single nucleotide polymorphisms (SNPs) that are strongly related to exposure (e.g. sedentary behavior) are used as instrumental variables (IVs) to estimate the causal effect with outcome (e.g. CKD and DM) [19]. It gets over the common problems with observational studies and allows the use of publicly available data from very large genome-wide association studies (GWAS) for both risk factor ‘exposures’ and disease ‘outcomes’[17]. All of the genetic variants must adhere to three IV assumptions: (1) genetic variants are strongly associated with exposure; (2) genetic variants influence outcome only by exposure; and (3) genetic variants are not affected by other confounding factors that affect the outcome [20]. Here, a two-sample Mendelian randomization analysis was performed to investigate sedentary behavior’s causal relationship in CKD, DM, and related complications.

## Methods

### CKD, DM, and related complications GWAS summary statistics

All GWAS summary statistics came from public databases to ensure accuracy and traceability and were supported by published articles (Table 1). Summary statistics of CKD are available in CKDGen Consortium (https://ckdgen.imbi.uni-freiburg.de/) including 480,698 European participants (41,395 cases and 439,303 controls), CKD defined as an estimated glomerular filtration rate (eGFR) lower than 60 mL^−1^ per 1.73 m^2^ [21]. GWAS data for type 1 diabetes (T1D) was from https://gwas.mrcieu.ac.uk/ (6447 cases and 451,248 controls) published in 2021 [22]. We downloaded CKD in T1D summary statistics from the Common Metabolic Diseases Knowledge Portal (CMDKP, https://hugeamp.org/); this study included 19,406 European T1D patients from 17 cohorts, different renal complications were covered, cases with eGFR below 60 mL^−1^ per 1.73 m^2^ were defined as CKD and controls were defined as eGFR > 60 mL^−1^ per 1.73 m^2^ accompanying history of diabetes for more than fifteen years [23]. T2D GWAS data were obtained from DIAbetes Genetics Replication And Meta-analysis (DIAGRAM) Consortium based on a T2D GWAS meta-analysis including 898,130 European participants (80,154 cases and 853,816 controls) [24]. Summary data for CKD in T2D was from a GWAS study of diabetic kidney disease, the definition of CKD was the same as CKDGen Consortium (6000 European cases) and controls had no CKD with duration of T2D more than 10 years [25]. Summary statistics of European DM patients (21,969 European ancestry cases, 433,048 European ancestry controls) were downloaded from GWAS Catalog (https://www.ebi.ac.uk/gwas/home) [26]. A GWAS meta-analysis with 10 renal complications phenotype among 27,000 diabetes patients proving the summary statistics of CKD in DM [27].

**Table 1.** Chronic kidney disease, diabetes, and related complications GWAS data source.

Traits	Sample size	Ancestry	Source
Chronic kidney disease	480,698	European	CKDGen Consortium https://ckdgen.imbi.uni-freiburg.de/ PMID:31152163
Type 1 Diabetes	520,580	European	IEU OpenGWAS project https://gwas.mrcieu.ac.uk/ PMID: 34594039
CKD in Type 1 Diabetes	19,406	European	Common Metabolic Diseases Knowledge Portal https://hugeamp.org/ PMID: 31537649
Type 2 Diabetes	898,130	European	DIAGRAM Consortium https://diagram-consortium.org/index.html PMID: 35551307
CKD in Type 2 Diabetes	6,000	European	GWAS Catalog https://www.ebi.ac.uk/gwas/home PMID:29703844
Diabetes Mellitus	455,017	European	GWAS Catalog https://www.ebi.ac.uk/gwas/home PMID: 34737426
CKD in Diabetes	27,000	European	Common Metabolic Diseases Knowledge Portal https://hugeamp.org/ PMID: 35763030

CKD: Chronic kidney disease; T1D: Type 1 Diabetes; T2D: Type 2 Diabetes; DM: Diabetes Mellitus.

### Sedentary behavior GWAS summary statistics

GWAS data for leisure sedentary behavior was from the latest GWAS meta-analysis including 422,218 European ancestry participants, in this study LSBs were divided into three categories containing leisure television watching, leisure computer use, and driving [12]. In this study, 45.7% of the participants were male, and the mean age at the time of the first assessment was 57.4 years (Standard Deviation (SD) 8.0). The average daily leisure time spent watching television was 2.8 h (SD 1.5), leisure time spent using a computer was 1.0 h (SD 1.2), and time spent driving was 0.9 h (SD 1.0) [12].

### Genetic instrumental variables

Instrumental variables (IVs) for LSBs used in this study were obtained from a published article which demonstrated the causal association between physical activity, LSB, and coronavirus disease 2019 (COVID-19) risk [28]. The screening process for IVs was divided into five steps, the first being the removal of linkage disequilibrium, the second being that only SNPs with F-statistics > 10 were selected to ensure the strength of the genetic tool, the third being to ensure that only SNPs with a genome-wide significance threshold of *p* < 5 × 10^−8^ were selected, the fourth being the exclusion of ambiguous and duplicated SNPs (effect allele frequency (EAF) > 0.42), and the fifth being the removal of SNPs with potentially pleiotropy by the MR-Pleiotropy RESidual Sum and Outlier (MR-PRESSO) method, and finally 84, 21, and 4 SNPs were used as IVs for leisure television watching, computer use, and driving, respectively [28]. IVs we used in this study were shown in the supplementary material Table S2.

### Mendelian randomization analysis

Genetic variants are used as IVs in MR analysis to determine the causal relationship between exposure and outcome. In the study, we combined the summary statistics to estimate sedentary behavior’s causal relationship in CKD, diabetes (including T1D, T2D, DM) and related complications (including CKD in T1D, CKD in T2D, and CKD in Diabetes) using different methods. Several reliable MR methods were applied to ensure the robustness of the results, including inverse variance weighted (IVW), MR-Egger, weighted median method (WMM), and MR-PRESSO methods.

IVW method, which combines the Wald ratio together, and the weight of each ratio is the inverse of the variance of the SNP-outcome association [29,30], was our principal method in this study. ${\hat{\beta }}_{\text{YZ}}$ and ${\hat{\beta }}_{\text{XZ}}$ are the coefficients from the regressions of the outcome and exposure, respectively, on the SNP instrument [31].

$${\hat{\gamma }}_{\text{Wald}}=\frac{{\hat{\beta }}_{\text{YZ}}}{{\hat{\beta }}_{\text{XZ}}}$$

Compared with IVW, the MR–Egger method adds an intercept item, and its main function is to test the horizontal pleiotropy; if the intercept term differs from zero, then the genetic variants are not all valid instruments, and the IVW estimates are biased [32]. When horizontal pleiotropy contributes at least half of the weighted variance, the WMM provides reliable effect estimates [33]. There are three types of detection functions of the MR-PRESSO method: horizontal pleiotropic detection, horizontal pleiotropic correction (after removing outliers), and whether there is a difference in the causality estimation results before and after the correction [34]. The MR-PRESSO outlier test depends on the Instrument Strength Independent of Direct Effect (InSIDE) condition, which states that pleiotropic effects and instrument exposure are uncorrelated, and that at least 50% of the variations are valid instruments [34].

After the Bonferroni correction for multiple testing, *p* value <0.002 (0.05/21, 3 exposures and 7 outcomes) was considered to be statistically significant.

### Sensitivity analysis

**Figure 1.**
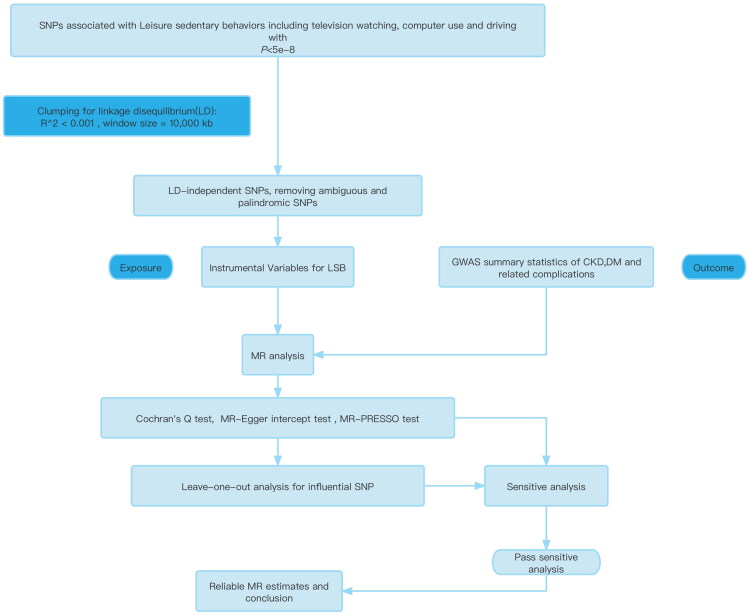
Flow chart about MR analysis.

When genetic variants associated with the exposure (sedentary behavior) did not affect the outcome (CKD, DM etc.) directly, horizontal pleiotropy occurs. Thus, we conducted the MR Egger regression and MR-PRESSO methods. When assessing heterogeneity in the IVW and MR-Egger regression, the Cochran’s Q test was used; a *p* value < 0.05 was regarded as statistically significant [30]. ‘Leave-one-out (LOO)’ analysis was used for detecting influential SNP. Flow chart about MR analysis performed step-by-step was shown in Figure 1.

A publicly accessible online database provided all the GWAS summary statistics. No further ethics approval or informed consent was required. Every participant provided written, informed consent, which was approved by the institutional ethical review boards involved in each study.

## Results

### Causal effects of LSB on CKD, DM, and related complications

**Figure 2.**
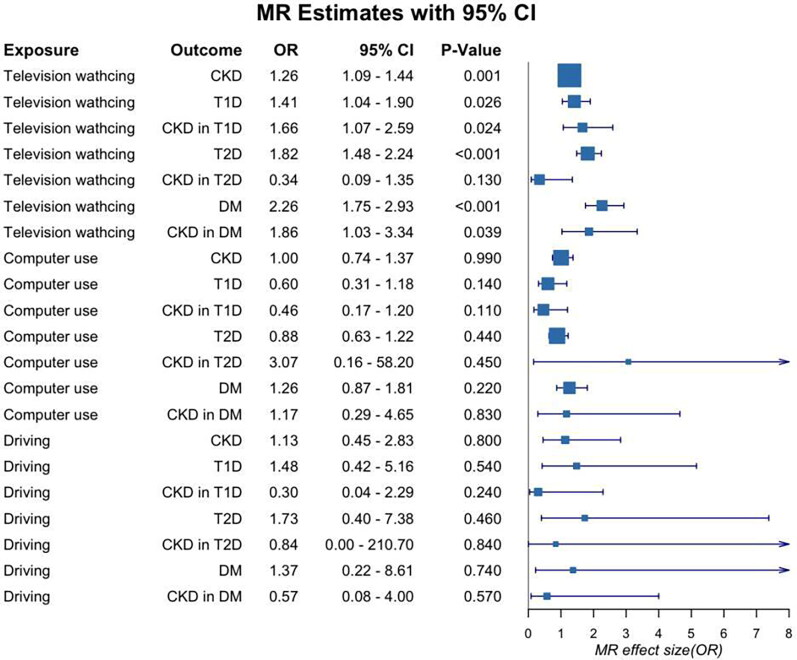
Forest plot for the effects of leisure sedentary behaviour on chronic kidney disease, diabetes and related complications.

MR estimates from several methods were used to evaluate the causal association between LSB and CKD, DM, and related complications (Supplement material Table S1). Figure 2 showed a forest plot of causal effects derived by the IVW method. According to the results, there was no causal effect between computer use and CKD, DM, and related complications, and neither was driving. Television watching had a harmful effect on CKD, T2D, and DM based on MR estimates. More television watching hours were associated with higher risk of CKD (IVW: OR = 1.26, 95%CI 1.09–1.44, *p* = 0.0011), T2D (IVW: OR = 1.82, 95%CI 1.48–2.24, *p* = 1.67 × 10−^8^) and DM (IVW: OR = 2.26, 95%CI 1.75–2.93; *p* = 6.44 × 10^−10^).

### Sensitivity analysis

Cochran’s Q test, MR-Egger intercept test, and MR-PRESSO test were applied for sensitivity analysis (Table 2). The MR-Egger intercept test demonstrated there was no horizontal pleiotropy. Heterogeneity was observed in Cochran’s Q test analysis (*p <* 0.05) but random effect model of IVW method was used in this study. There was no significant distortion after removal of outliers in MR-PRESSO test and we did not find influential SNPs in ‘leave-one-out’ analysis. Scatter plots and ‘leave-one-out’ plots were combined in Figure 3.

Figure 3.Scatter plots and “leave one out” analysis plots for MR analysis of causal association of television watching on chronic kidney disease, diabetes and related complications. a, CKD. b, T1D. c, CKD in T1D. d, T2D. e, CKD in T2D. f, DM. g, CKD in DM.

**Figure:**
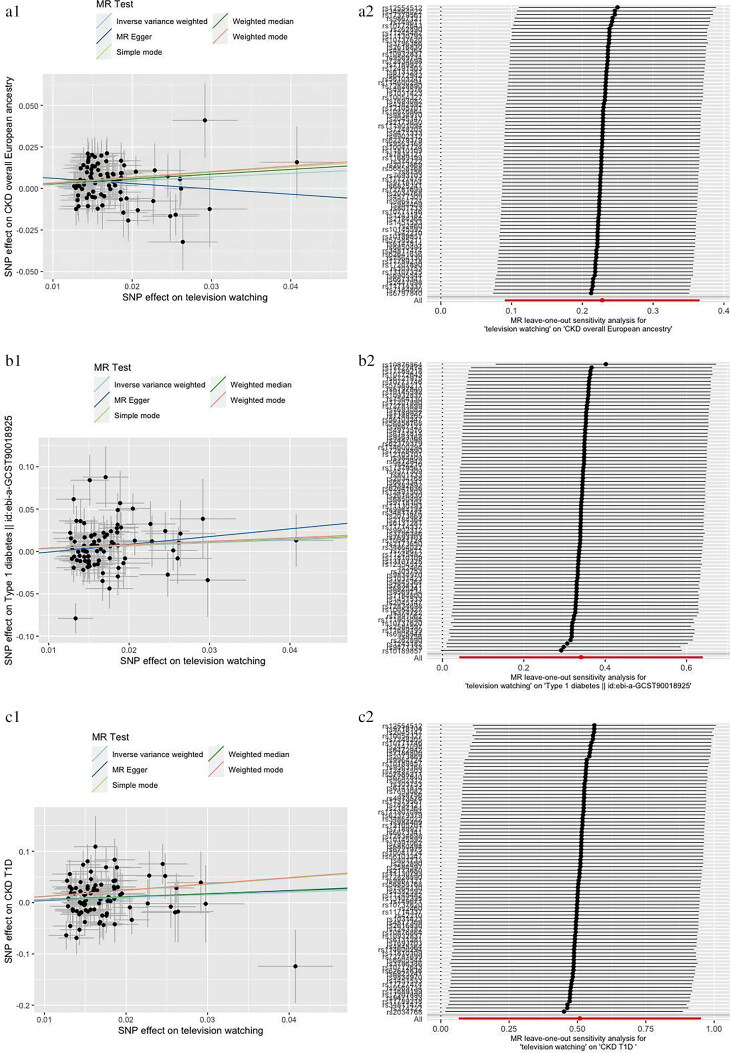

**Figure:**
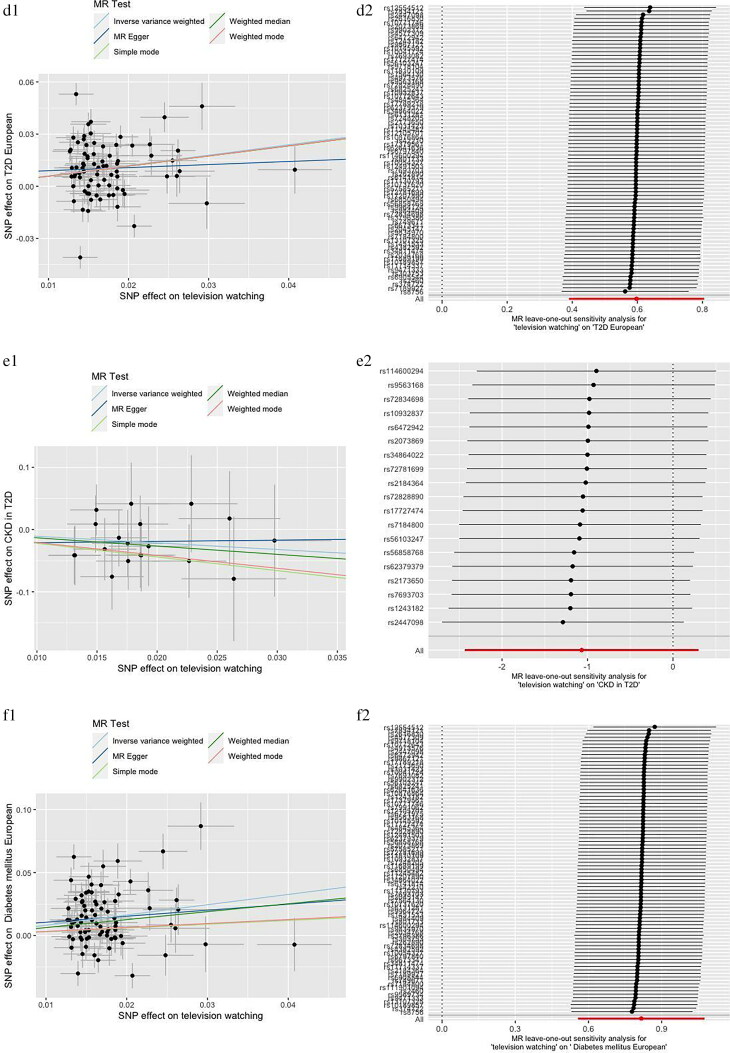

**Figure:**
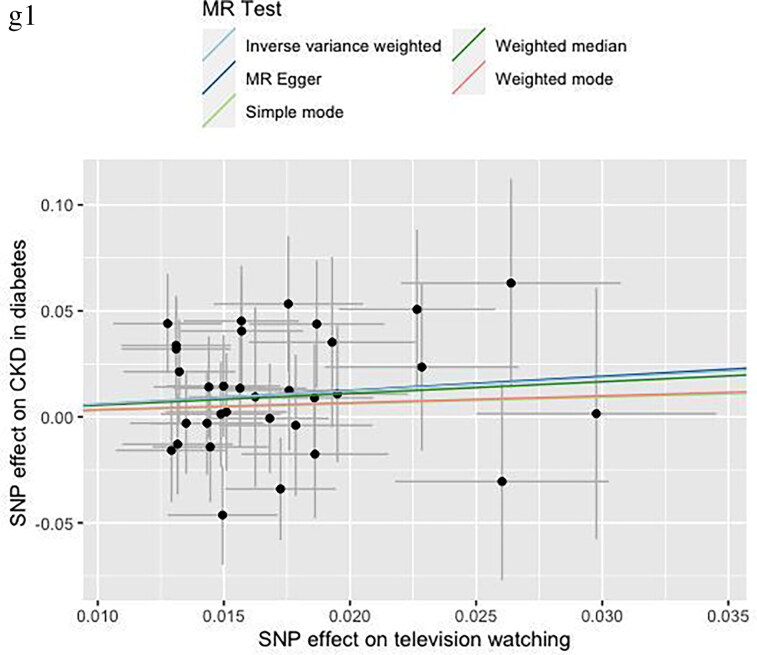

**Table 2.** Sensitive analysis results for MR estimates of television watching on outcomes.

Outcome	Cochran Q test		MR Egger		MR-PRESSO distortion Test
	Q value	*p*	Intercept	*p*	
CKD	77.52	0.56	0.0094	0.11	0.55
T1D	112.75	0.017	−0.0099	0.42	0.70
CKD in T1D	94.0	0.19	−0.0025	0.90	*NA*
T2D	367.01	2.30e-39	0.0072	0.40	0.73
CKD in T2D	7.20	0.99	−0.023	0.72	*NA*
DM	265.82	2.89e-21	0.0058	0.59	0.68
CKD in DM	28.12	0.66	−0.00073	0.98	0.67

The MR-Egger method accounts for horizontal pleiotropy, which may lead to different estimates compared to other MR approaches. In our study, the MR-Egger intercept test did not indicate significant pleiotropy (*p* = 0.11), suggesting that the observed difference is more likely due to the high variability in MR-Egger estimates rather than true pleiotropic effects. Additionally, MR-Egger often has lower precision due to its reliance on fewer valid instruments, which can result in a directionally different point estimate.

## Discussion

Using different large-scale GWAS data from the public online databases, this study implemented diverse MR methods to assess the potential causal association of sedentary behavior with CKD, DM, and related complications in the European population. Our analysis differentiates the effects of three types of LSB—television watching, computer use, and driving—demonstrating that television watching is the most strongly associated with CKD, T2D, and DM, while computer use and driving did not show significant causal effects. We demonstrated that television watching causally increased the risk for CKD, T1D, T2D, DM, CKD in T1D and CKD in DM. After the Bonferroni correction, television watching causally increased the risk for CKD, T2D, and DM. In view of the soaring years of life losses of CKD, diabetes and related complications in society, our study provides novel insight into reducing the risk of above diseases by highlighting the importance of physical activities in daily life. The observed variations in the effects of television watching, computer use, and driving on kidney disease and diabetes may be influenced by differences in activity intensity, duration, and their interactions with other lifestyle factors, such as diet and physical exercise[35–38].

A prolonged sedentary lifestyle has been linked to higher chances of all-cause mortality and a number of significant noncommunicable illnesses[39]. Patients with CKD-suffered anemia, reduction in the function of heart and skeletal muscle, and poor exercise tolerance, which made it harder to carry out everyday tasks and increase the sedentary time[40]. Even after accounting for moderate-to-vigorous physical activity (MVPA), epidemiological research, primarily involving younger and middle-aged persons, has consistently and strongly linked self-reported TV time to incident diabetes[41]. When compared to comparatively unworkable large-scale prospective clinical trials that necessitate long-term follow-up, the MR study quickly and affordably provides new insight into the causal link between LSB and CKD, DM and related complications.

Television watching was the most typical leisure sedentary activity when compared to other sedentary features[16]. It caused the energy balance to move toward an energy surplus by reducing breaks, lowering energy expenditure, and consuming excessive amounts of energy (particularly snacks)[42]. Our study found that television watching causally increased the risk for CKD, T2D, and DM. Decreased activity and increased sedentary behavior (television watching) lead to decreased renal function, which is consistent with most clinical studies. A study from the Quebec Adipose and Lifestyle Investigation in Youth (QUALITY) cohort of children of Western European descent supported sedentary behavior as a major contributor to the development of T2D in children and adolescents at risk for the disease was reinforced by the use of contemporary causal inference techniques, and these behaviors should be prioritized for prevention [43]. As for T2D adults, an MR study showed less TV watching was related to a decreased T2DM risk [44], which was consistent with our study. Meanwhile, researchers used the Objective Physical Activity and Cardiovascular Disease Health in Older Women (OPACH) Study statistically replacing sedentary time with MVPA, and found it was associated with lower diabetes risk in older women [45]. However, in T1DM and the whole DM patients, relevant studies only focused on its correlation with psychological well-being [46]. In view of the possibility of bias due to clinical research methods, study cohort, population, analysis strategy and so on, our study uses MR methods to explore the causal relationship between LSB and CKD, diabetes and related complications. We found LSB related to T2D and DM but not T1D was probably due to the mediating effect and the overlap between populations present in T2D and DM.

*Kidney disease*: Clinical practice guidelines by the Improving Global Outcomes (KDIGO) diabetes Working group, 2022, for the management of diabetes in chronic kidney disease, pointed out that patients with diabetes and chronic kidney disease should focus on lifestyle intervention [47]. Recommend that people with diabetes and chronic kidney disease engage in at least 150 min of moderate-intensity physical activity weekly or at a level appropriate for their cardiovascular and physical tolerance (1D) [47]. Combined with the conclusions of our study on causality, the formulation of appropriate exercise prescription is particularly important for the occurrence of related diseases.

However, there are several limitations in our study. First, only European population GWAS summary statistics were used in this study; thus, the conclusion does not apply to other populations. Second, it was difficult to completely distinguish between mediation in this MR study. To further substantiate the metabolic pathways underlying the causal association between television watching and CKD, DM and related complications, more observational studies and mediator analyses are needed. Future research should consider age as a potential confounding factor, as older individuals may be more susceptible to the harmful effects of prolonged sedentary behavior due to age-related declines in kidney function and physical activity levels.

## Conclusions

Using comprehensive genetic summary data, this study first comprehensively analyzes the causal relationship between LSB and CKD, DM and related complications. We found that more television watching was related to increased risk of CKD, T2D, and DM. It is necessary to do more research to understand the underlying mechanisms between them. With the highest cause of years of life loss of CKD and DM, lifestyle management should receive a lot of attention.

## Supplementary Material

Supplemental Material

Supplemental Material

## Data Availability

All GWAS summary statistics came from public database to ensure accuracy and traceability and were supported by published articles. Summary statistics of CKD are available in CKDGen Consortium (https://ckdgen.imbi.uni-freiburg.de/). We downloaded CKD in T1D summary statistics from the Common Metabolic Diseases Knowledge Portal (CMDKP, https://hugeamp.org/). T2D GWAS data was obtained from DIAbetes Genetics Replication and Meta-analysis (DIAGRAM) Consortium. Summary statistics of European DM patients were downloaded from GWAS Catalog (https://www.ebi.ac.uk/gwas/home). GWAS data for leisure sedentary behavior was from latest GWAS meta-analysis (DOI: 10.1038/s41467-020-15553-w).
