# Supplementary material for: The impact of leisure sedentary behaviors on risk of chronic kidney disease, diabetes, and related complications: Mendelian randomization study
Source: Ren Fail. 2025 Mar 20;47(1):2479177. doi: 10.1080/0886022X.2025.2479177 (PMC11926908; doi:10.1080/0886022X.2025.2479177)
Supplement: Supplemental Material [file IRNF_A_2479177_SM8351.docx]

**Table S1** MR analysis results of assessing the causal association of leisure sedentary behaviour on outcomes

| Outcome | IVW/Wald ratio | | MR Egger | | Weighted Median | |
| --- | --- | --- | --- | --- | --- | --- |
|  | OR 95%CI | P value | OR 95%CI | P value | OR 95%CI | P value |
| ***Television Watching*** | | | | | | |
| CKD | 1.26  (1.09-1.44） | 0.0011 | 0.73  (0.37-1.42) | 0.35 | 1.33  (1.08-1.64) | 0.0064 |
| T1D | 1.41  (1.04-1.90) | 0.026 | 2.50  (0.61- 10.30) | 0.21 | 1.45  (0.98-2.13) | 0.06 |
| CKD in T1D | 1.66  (1.07-2.59) | 0.024 | 1.93  (0.20-18.24) | 0.57 | 1.78  0.99-3.19 | 0.053 |
| T2D | 1.82  (1.48-2.24) | 1.67e-08 | 1.19  (0.44-3.22) | 7.27e-01 | 1.79  (1.5- 2.12) | 2.50e-11 |
| CKD in T2D | 0.34  (0.088-1.35) | 0.13 | 1.24  (0.0012-1253.13) | 0.95 | 0.26  (0.043-1.65) | 0.14 |
| DM | 2.26  (1.75-2.93) | 6.44e-10 | 1.61  (0.45-5.73) | 4.63e-01 | 1.88  (1.47-2.40) | 3.84e-07 |
| CKD in DM | 1.86  (1.03-3.34) | 0.039 | 1.94  (0.083-45.60) | 0.68 | 1.74  (0.75-4.00) | 0.19 |

***Computer Use***

| CKD | 1.003  (0.74-1.37) | 0.99 | 0.26  (0.02-2.79) | 0.28 | 0.82  (0.55-1.23) | 0.34 |
| --- | --- | --- | --- | --- | --- | --- |
| T1D | 0.60  (0.31-1.18) | 0.14 | 0.47  (0.002-94.17) | 0.78 | 0.43  (0.20-0.93) | 0.03 |
| CKD in T1D | 0.46  (0.17-1.20) | 0.11 | 6.77  (0.003-13419.32) | 0.63 | 0.33  (0.10-1.09) | 0.07 |
| T2D | 0.88  (0.63-1.22) | 0.44 | 2.53  (0.20-32.37) | 0.49 | 0.80  (0.58-1.10) | 0.16 |
| CKD in T2D | 3.07  (0.16-58.2) | 0.45 | 0.03  (1.25e-36-5.46e+32) | 0.94 | 3.42  ( 1.11e-01-1.05e+02) | 0.48 |
| DM | 1.26  (0.87-1.81) | 0.22 | 2.60  (0.14-49.72) | 0.53 | 1.15  (0.73-1.81) | 0.54 |
| CKD in DM | 1.17  (0.29-4.65) | 0.83 | 75.52  (0.0014-4.01e+06) | 0.46 | 1.54  (0.37-6.41) | 0.55 |

***Driving***

| CKD | 1.13  (0.45-2.83) | 0.80 | 0.007  (1.44e-05 3.44) | 0.26 | 1.05  (4.71e-01-2.34) | 0.91 |
| --- | --- | --- | --- | --- | --- | --- |
| T1D | 1.48  (0.42-5.16) | 0.54 | 1.83  (5.43e-05 61658.44) | 0.92 | 1.09  (0.25-4.74) | 0.91 |
| CKD in T1D | 0.30  (0.039-2.29) | 0.24 | 0.98  (2.22e-08-4.34e+07) | 0.998 | 0.28  (2.88e-02-2.75) | 0.28 |
| T2D | 1.73  (0.40-7.38) | 0.46 | 0.91  (4.12e-07- 2.0e+06) | 0.91 | 2.13  (1.12-4.06) | 0.02 |
| CKD in T2D | 0.84  (0.001-210.7) | 0.84 | *NA* | *NA* | *NA* | *NA* |
| DM | 1.37  (0.22-8.61) | 0.74 | 0.027  (6.19e-10-1.14e+06) | 0.73 | 1.33  (0.49-3.64) | 0.58 |
| CKD in DM | 0.57  (0.082-4.0) | 0.57 | 0.15  (7.66e-08-2.96e+05) | 0.84 | 0.50  (0.054-4.55) | 0.54 |
