# Supplementary material for: The impact of leisure sedentary behaviors on risk of chronic kidney disease, diabetes, and related complications: Mendelian randomization study
Source: Ren Fail. 2025 Mar 20;47(1):2479177. doi: 10.1080/0886022X.2025.2479177 (PMC11926908; doi:10.1080/0886022X.2025.2479177)
Supplement: Supplemental Material [file IRNF_A_2479177_SM7911.docx]

**Table S2** Statistical details of SNPs as instrumental variable

| No. | SNP | A1 | A2 | EAF | β | SE | P | F-statistics |
| --- | --- | --- | --- | --- | --- | --- | --- | --- |
| 1 | rs10041724 | T | C | 0.808 | 0.0181 | 0.0027 | 1.96E-11 | 43.65 |
| 2 | rs10054327 | G | A | 0.576 | 0.0172 | 0.0022 | 1.72E-15 | 61.99 |
| 3 | rs10145592 | C | G | 0.409 | -0.0148 | 0.0022 | 8.89E-12 | 45.20 |
| 4 | rs10189857 | A | G | 0.568 | -0.0205 | 0.0022 | 3.09E-21 | 88.11 |
| 5 | rs1031423 | T | C | 0.215 | -0.0185 | 0.0026 | 8.82E-13 | 49.73 |
| 6 | rs10737620 | T | A | 0.274 | 0.0144 | 0.0024 | 1.28E-09 | 35.50 |
| 7 | rs10771746 | C | T | 0.717 | -0.0143 | 0.0024 | 1.23E-09 | 35.57 |
| 8 | rs10772643 | C | T | 0.108 | 0.0248 | 0.0035 | 6.58E-13 | 50.31 |
| 9 | rs10876864 | G | A | 0.427 | -0.0133 | 0.0022 | 5.07E-10 | 37.30 |
| 10 | rs10932837 | C | T | 0.494 | -0.0131 | 0.0022 | 6.68E-10 | 36.76 |
| 11 | rs11130793 | C | T | 0.602 | 0.0129 | 0.0022 | 2.52E-09 | 34.17 |
| 12 | rs111901094 | G | T | 0.817 | -0.0171 | 0.0028 | 9.91E-10 | 35.99 |
| 13 | rs11245482 | T | C | 0.613 | -0.0132 | 0.0022 | 1.28E-09 | 35.49 |
| 14 | rs114600294 | G | C | 0.789 | -0.0162 | 0.0026 | 3.94E-10 | 37.79 |
| 15 | rs11689199 | A | G | 0.598 | 0.0185 | 0.0022 | 2.74E-17 | 70.16 |
| 16 | rs11714337 | G | A | 0.569 | 0.0144 | 0.0022 | 2.36E-11 | 43.29 |
| 17 | rs11810109 | A | T | 0.701 | 0.0163 | 0.0024 | 2.69E-12 | 47.54 |
| 18 | rs12105701 | C | T | 0.396 | -0.0129 | 0.0022 | 2.72E-09 | 34.03 |
| 19 | rs1243182 | C | T | 0.690 | -0.0186 | 0.0023 | 1.01E-15 | 63.04 |
| 20 | rs12491503 | G | A | 0.670 | -0.0143 | 0.0023 | 2.77E-10 | 38.48 |
| 21 | rs12554512 | T | C | 0.584 | 0.0207 | 0.0022 | 1.88E-21 | 89.09 |
| 22 | rs13107325 | C | T | 0.926 | -0.0292 | 0.0041 | 7.43E-13 | 50.07 |
| 23 | rs1451533 | G | A | 0.725 | -0.0157 | 0.0024 | 5.43E-11 | 41.66 |
| 24 | rs17207890 | G | A | 0.657 | 0.0157 | 0.0023 | 3.36E-12 | 47.10 |
| 25 | rs17379561 | A | T | 0.856 | -0.0255 | 0.0031 | 5.41E-17 | 68.81 |
| 26 | rs17727474 | C | T | 0.832 | 0.0176 | 0.0030 | 1.54E-09 | 35.14 |
| 27 | rs17789218 | T | C | 0.756 | 0.0186 | 0.0025 | 6.96E-14 | 54.72 |
| 28 | rs2034768 | A | G | 0.487 | 0.0147 | 0.0022 | 4.31E-12 | 46.62 |
| 29 | rs2045147 | A | G | 0.449 | 0.0127 | 0.0022 | 2.94E-09 | 33.88 |
| 30 | rs2073869 | C | T | 0.834 | 0.0186 | 0.0029 | 7.72E-11 | 40.97 |
| 31 | rs2173650 | G | T | 0.852 | 0.0178 | 0.0030 | 2.28E-09 | 34.37 |
| 32 | rs2184364 | A | G | 0.782 | 0.0156 | 0.0026 | 1.50E-09 | 35.18 |
| 33 | rs2447098 | C | A | 0.475 | -0.0149 | 0.0022 | 3.41E-12 | 47.08 |
| 34 | rs2460 | G | A | 0.736 | -0.0153 | 0.0025 | 2.48E-10 | 38.69 |
| 35 | rs2584597 | T | C | 0.662 | 0.0151 | 0.0024 | 1.46E-10 | 39.72 |
| 36 | rs2616830 | G | A | 0.462 | 0.0165 | 0.0022 | 1.44E-14 | 57.81 |
| 37 | rs262890 | A | G | 0.699 | -0.0186 | 0.0024 | 1.60E-15 | 62.13 |
| 38 | rs303753 | G | A | 0.653 | -0.0145 | 0.0023 | 1.37E-10 | 39.85 |
| 39 | rs34811474 | G | A | 0.768 | 0.0153 | 0.0026 | 1.14E-09 | 35.72 |
| 40 | rs34864022 | A | G | 0.934 | -0.0264 | 0.0044 | 7.03E-10 | 36.66 |
| 41 | rs374722 | G | A | 0.150 | 0.0245 | 0.0030 | 2.74E-16 | 65.61 |
| 42 | rs3796386 | G | A | 0.572 | -0.0262 | 0.0022 | 1.61E-33 | 144.19 |
| 43 | rs42210 | G | C | 0.289 | -0.0139 | 0.0024 | 3.68E-09 | 33.44 |
| 44 | rs4382592 | T | G | 0.301 | 0.0137 | 0.0024 | 3.34E-09 | 33.63 |
| 45 | rs4577309 | A | G | 0.469 | 0.0160 | 0.0022 | 7.80E-14 | 54.49 |
| 46 | rs4845364 | A | G | 0.495 | -0.0153 | 0.0022 | 6.39E-13 | 50.36 |
| 47 | rs4973576 | C | A | 0.298 | -0.0145 | 0.0024 | 5.19E-10 | 37.25 |
| 48 | rs56103247 | C | T | 0.944 | 0.0298 | 0.0048 | 1.89E-10 | 39.23 |
| 49 | rs56858768 | G | A | 0.701 | -0.0149 | 0.0024 | 1.70E-10 | 39.43 |
| 50 | rs57585211 | T | G | 0.827 | -0.0167 | 0.0029 | 2.16E-09 | 34.47 |
| 51 | rs6131281 | C | T | 0.597 | 0.0161 | 0.0022 | 1.54E-13 | 53.16 |
| 52 | rs6141814 | C | A | 0.613 | -0.0135 | 0.0022 | 6.55E-10 | 36.80 |
| 53 | rs62379379 | G | T | 0.929 | -0.0260 | 0.0042 | 3.89E-10 | 37.81 |
| 54 | rs62641636 | A | G | 0.692 | 0.0144 | 0.0023 | 3.49E-10 | 38.02 |
| 55 | rs6472942 | T | C | 0.568 | -0.0132 | 0.0022 | 8.87E-10 | 36.21 |
| 56 | rs6673341 | T | G | 0.465 | -0.0145 | 0.0022 | 1.11E-11 | 44.76 |
| 57 | rs6721975 | T | C | 0.232 | -0.0167 | 0.0026 | 8.57E-11 | 40.77 |
| 58 | rs6797840 | A | C | 0.456 | -0.0161 | 0.0022 | 8.35E-14 | 54.36 |
| 59 | rs6825241 | C | A | 0.536 | -0.0170 | 0.0022 | 2.46E-15 | 61.29 |
| 60 | rs6850494 | A | C | 0.616 | -0.0143 | 0.0022 | 5.67E-11 | 41.58 |
| 61 | rs6905544 | A | G | 0.399 | -0.0190 | 0.0022 | 4.23E-18 | 73.84 |
| 62 | rs7184800 | G | A | 0.697 | 0.0168 | 0.0023 | 4.09E-13 | 51.24 |
| 63 | rs7189927 | T | C | 0.356 | 0.0150 | 0.0023 | 1.68E-11 | 43.95 |
| 64 | rs7248205 | C | T | 0.398 | 0.0139 | 0.0022 | 1.68E-10 | 39.46 |
| 65 | rs72781699 | G | A | 0.797 | -0.0187 | 0.0027 | 1.52E-12 | 48.66 |
| 66 | rs72828890 | C | T | 0.869 | 0.0193 | 0.0033 | 2.47E-09 | 34.21 |
| 67 | rs72834698 | G | A | 0.858 | 0.0227 | 0.0031 | 1.37E-13 | 53.39 |
| 68 | rs749671 | G | A | 0.629 | 0.0157 | 0.0022 | 1.34E-12 | 48.92 |
| 69 | rs7564130 | T | C | 0.641 | -0.0150 | 0.0023 | 1.38E-11 | 44.34 |
| 70 | rs7693082 | G | C | 0.300 | 0.0151 | 0.0024 | 8.27E-11 | 40.84 |
| 71 | rs7693703 | G | A | 0.910 | 0.0228 | 0.0038 | 1.37E-09 | 35.37 |
| 72 | rs7834121 | G | T | 0.504 | -0.0140 | 0.0022 | 5.53E-11 | 41.62 |
| 73 | rs7991062 | C | G | 0.659 | -0.0177 | 0.0023 | 4.41E-15 | 60.14 |
| 74 | rs801733 | A | C | 0.641 | 0.0169 | 0.0023 | 3.66E-14 | 55.98 |
| 75 | rs8756 | C | A | 0.485 | -0.0135 | 0.0022 | 2.66E-10 | 38.55 |
| 76 | rs9471333 | C | T | 0.450 | 0.0131 | 0.0022 | 7.69E-10 | 36.49 |
| 77 | rs9563168 | G | A | 0.791 | 0.0176 | 0.0027 | 2.15E-11 | 43.47 |
| 78 | rs9569734 | A | G | 0.844 | 0.0189 | 0.0030 | 1.69E-10 | 39.44 |
| 79 | rs9718104 | T | G | 0.942 | -0.0408 | 0.0046 | 4.65E-19 | 78.20 |
| 80 | rs9834970 | T | C | 0.501 | 0.0128 | 0.0022 | 1.63E-09 | 35.02 |
| 81 | rs984409 | G | A | 0.363 | -0.0149 | 0.0023 | 2.06E-11 | 43.55 |
| 82 | rs9867121 | C | A | 0.818 | 0.0195 | 0.0028 | 1.94E-12 | 48.19 |
| 83 | rs9902312 | T | C | 0.683 | 0.0153 | 0.0023 | 2.27E-11 | 43.36 |
| 84 | rs9964724 | C | T | 0.318 | 0.0176 | 0.0023 | 1.67E-14 | 57.53 |
